# Yohimbine Enhances Protection of Berberine against LPS-Induced Mouse Lethality through Multiple Mechanisms

**DOI:** 10.1371/journal.pone.0052863

**Published:** 2012-12-28

**Authors:** Hui Li, Yiyang Wang, Haoqing Zhang, Baoyin Jia, Daan Wang, Hongmei Li, Daxiang Lu, Renbin Qi, Yuxia Yan, Huadong Wang

**Affiliations:** 1 Department of Pathophysiology, Key Laboratory of State Administration of Traditional Chinese Medicine of the People’s Republic of China, School of Medicine, Jinan University, Guangzhou, China; 2 Department of Otolaryngology, Guangzhou Overseas Chinese Hospital, School of Medicine, Jinan University, Guangzhou, China; 3 Department of Biochemistry, School of Medicine, Jinan University, Guangzhou, China; 4 Center of Prenatal Diagnosis, Chenzhou No. 1 People’s Hospital, Chenzhou, Hunan, China; University Hospital Freiburg, Germany

## Abstract

Sepsis remains a major cause of mortality in intensive care units, better therapies are urgently needed. Gram-negative bacterial lipopolysaccharide (LPS) is an important trigger of sepsis. We have demonstrated that berberine (Ber) protects against lethality induced by LPS, which is enhanced by yohimbine (Y) pretreatment, and Ber combined with Y also improves survival in septic mice. However, the precise mechanisms by which Y enhances protection of Ber against LPS - induced lethality remain unclear. The present study confirmed that simultaneously administered Y also enhanced protection of Ber against LPS-induced lethality. Ber or/and Y attenuated liver injury, but not renal injury in LPS-challenged mice. Ber or/and Y all inhibited LPS-stimulated IκBα, JNK and ERK phosphorylation, NF-κB activation as well as TNF-α production. Ber also increased IL-10 production in LPS-challenged mice, which was enhanced by Y. Furthermore, Ber or/and Y all suppressed LPS-induced IRF3, TyK2 and STAT1 phosphorylation, as well as IFN-β and IP-10 mRNA expression in spleen of mice at 1 h after LPS challenge. Especially, Y enhanced the inhibitory effect of Ber on LPS-induced IP-10 mRNA expression. *In vitro* experiments further demonstrated that Y significantly enhanced the inhibitory effect of Ber on TNF-α production in LPS-treated peritoneal macrophages, Ber combined with Y promoted LPS-induced IL-10 production and LPS-stimulated IκBα, JNK, ERK and IRF3 phosphorylation and NF-κB activation were also suppressed by Ber or/and Y pretreatment in peritoneal macrophages. Taken together, these results demonstrate that Y enhances the protection of Ber against LPS-induced lethality in mice via attenuating liver injury, upregulating IL-10 production and suppressing IκBα, JNK, ERK and IRF3 phosphorylation. Ber combined with Y may be an effective immunomodulator agent for the prevention of sepsis.

## Introduction

Sepsis is a systemic host response to an infection, characterized by a misbalance between proinflammatory reactions and anti-inflammatory responses [1]. Despite tremendous basic research advances and clinical efforts, severe sepsis remains a leading cause of mortality in the intensive care unit with its occurrence been increasing [2]. Recently, it was reported that the incidences of sepsis and septic shock exceeded those of myocardial infarction in general-surgery patients in the United States [3]. Therefore, it is very important to develop new therapies for improving survival in septic patients. A substantial body of experimental and clinical evidence demonstrates that bacterial products, such as lipopolysaccharide (LPS) and superantigens, as well as endogenous proinflammatory cytokines play a critical role in the pathogenesis of sepsis-induced organ failure [4], [5]. LPS, sensed by Toll-like receptor 4 (TLR4) on immune cells, activates the inhibitors of κB (IκB) kinase complex and mitogen-activated protein kinase (MAPK) kinases via myeloid differentiation factor 88 (MyD88)-dependent pathway, resulting in phosphorylation of IκB, extracellular signal-regulated kinase 1/2 (ERK1/2), p38 MAPK and c-Jun N-terminal kinases (JNK) and the subsequent activation of the transcription factor nuclear factor-κB (NF-κB) and activator protein-1 (AP-1), which induce the expression of cytokines such as tumor necrosis factor-α (TNF-α), interleukin (IL)-1β, IL-12, IL-10 and interferon-γ (IFN-γ). LPS also induces the phosphorylation of IFN regulatory factor 3 (IRF3), which stimulates expression of IFN-β, through MyD88-independent pathway. The produced IFN-β causes the phosphorylation of tyrosine kinase 2 (Tyk2) and signal transducer and activator of transcription 1 (STAT1), leading to the inducible nitric oxide synthase (iNOS) and IFN-inducible protein-10 (IP-10) expression [6]–[8]. This progressive release of cytokines and other inflammatory mediators, and severe dysregulation of the inflammatory network eventually lead to the impairment of host defence response, tissue damage, multiple organ dysfunction and even death 9. Thus, the development of novel immunomodulatory strategies to antagonize LPS-induced lethality is important to the treatment of sepsis, especially Gram-negative sepsis.

LOOSESTBerberine (Ber), a natural isoquinoline alkaloid, was reported to modulate cytokine expression and improve survival in endotoxemic mice [10]. Kang et al. observed that Ber induced p38 MAPK phosphorylation and then stimulated IL-12 p40 production via activating α_2_-adrenergic receptor in mouse macrophages [11]. We found that Ber attenuates LPS-induced lung injury through α_2_-adrenoceptor-independent mechanisms [12]. Furthermore, pretreatment with Ber protected against the lethality induced by LPS, which was enhanced by blocking α_2_-adrenergic receptor with yohimbine (Y), an α_2_-adrenergic receptor antagonist, and Ber combined with Y also increased survival rate in mice with sepsis induced by cecal ligation and puncture (CLP) [12]. However, the precise mechanisms by which Y enhances protection of Ber against LPS - induced mouse lethality remains unclear. In this study, we further investigated the effects of Ber and Y on cytokine expression, MyD88-dependent and independent signaling pathway activation in mice and mouse peritoneal macrophages challenged with LPS. The results suggest that Y enhances protection of Ber against LPS - induced mouse lethality via inhibiting IκBα, JNK and ERK phosphorylation as well as MyD88-independent signaling pathway activation, upregulating IL-10 expression and attenuating hepatic injury.

## Materials and Methods

### Mice

Male BALB/c mice (6–8 weeks old) were purchased from the medical laboratory animal center of Guangdong province (Guangzhou, China) and allowed to acclimate to the new environment for 3–4 days prior to experiment in a standard experimental room (12 h light/dark cycle, 24°C and 50%–70% humidity). Commercial standard food and tap water were provided *ad libitum*. All experiments were conducted in accordance with the Guide for the Care and Use of Laboratory Animals published by the US National Institutes of Health (NIH Publication No. 85–23, revised 1996), and the animal experimental procedures were previously approved by the Animal Care and Use Committee at School of Medicine, Jinan University. All surgery was performed under sodium pentobarbital anesthesia, and every effort was made to minimize suffering.

### Reagents and Antibodies

LPS (*Escherichia coli* serotype 055:B5), Ber hemisulfate and yohimbine (Y) were obtained from Sigma-Aldrich Co. (St. Louis, MO, USA). Enzyme-linked immunosorbent assay (ELISA) kits for the detection of mouse TNF-α, IL-1β, IL-12p40, IFN-γ and IL-10 were purchased from R&D Systems (Minneapolis, MN, USA). Antibodies, including IκBα, ERK, JNK, p38MAPK, NF-κB, IRF3, Tyk2, STAT1, phosphorylated IκBα (p-IκBα), phosphorylated JNK (p-JNK), phosphorylated ERK(p-ERK), phosphorylated p38MAPK (p-p38 MAPK), phosphorylated IRF3(p-IRF3), phosphorylated Tyk2 (p-Tyk2), phosphorylated STAT1(p-STAT1) and glyceraldehyde 3-phosphate dehydrogenase (GAPDH), and horseradish peroxidase -labeled secondary antibody were from Cell Signaling Technology, Inc. (Danvers, MA, USA).

### Treatment Protocols

In survival study, male BALB/c mice were randomly divided into following groups: control, LPS, Ber+LPS, Y+Ber+LPS, Y+LPS, Ber, Y+Ber and Y. The mice were treated intragastrically with distilled water (0.1 mL/10 g), Ber (50 mg/kg), Ber (50 mg/kg) in combination with Y (2 mg/kg) or Y (2 mg/kg) once a day for 3 days. One hour after last intragastric treatment on day 3, LPS (20 mg/kg, 0.2 mL/10 g body weight) or normal saline (0.2 mL/10 g body weight) was injected intraperitoneally. Survival was monitored every 12 h during the next 6 days. In parallel experiments, mice were treated as mentioned above. At indicated time, the mice were anesthetized with pentobarbital (100 mg/kg, i.p.) and sacrificed, blood samples were taken for measurement of cytokines, nitric oxide (NO) and biochemical values relevant to liver or kidney function, hepatic and renal tissues were obtained for histology, and the spleen was frozen in liquid nitrogen for RNA and protein analysis.

### Measurement of NO, Alanine Aminotransferase (ALT) and Blood Urea Nitrogen (BUN) Levels in Plasma

Plasma ALT and BUN levels were detected with an autoanalyzer (Vitros 750, Johnson-Johnson Co., NY). The concentration of nitrite (an indicator of NO) in the plasma was quantified by the method of Griess reaction according to the manufacturer’s specifications.

### Histological Analysis

Liver and renal tissues from different groups were removed at 12 h post LPS challenge, fixed in 10% phosphate-buffered formaldehyde, dehydrated in graded ethanol, embedded in paraffin, and prepared for histological slides. Slides were stained with hematoxylin & eosin (H&E).

### Mouse Peritoneal Macrophage Culture and Treatment

Peritoneal macrophages were obtained from 6 to 8 week-old male BALB/c mice as described previously [13]. In brief, macrophages were collected by washing the peritoneal cavity with cold phosphate-buffered saline (PBS) and incubated in RPMI-1640 medium (containing 20 mM Hepes, 2 mM glutamine, 100 µg/ml streptomycin, 100 U/ml penicillin and 10% FBS, pH 7.2) at 37°C for 6 h in a humified CO_2_ incubator. The cultured peritoneal macrophages (1×10^5^/ml) were treated with vehicle, Ber or/and Y for 2 h, and then exposed to 100 ng/ml LPS or normal saline for another 1 h or 6 h. Cell viability was measured with the Cell Counting kit-8 (Dojindo Molecular Technologies, Inc., Japan) as the manufacturer’s instructions. At 6 h after LPS treatment, TNF-α and IL-10 concentrations in the supernatants were examined. In the separate experiments, phosphorylation of IκBα, ERK, JNK, p38 and IRF3 as well as NF-κB activity in macrophages at 1 h after LPS challenge were analyzed.

### Measurement of Cytokines

Plasma and culture supernatants of peritoneal macrophages were collected at indicated time, concentrations of TNF-α, IL-1β, IL-12p40, IFN-γ and IL-10 were measured by using a commercially available ELISA kit according to the manufacturer’s instructions.

### Western Blotting

Spleen was removed 1 h after LPS insult, and homogenized in RIPA buffer (PBS, 1% NP-40, 0.5% sodium deoxycholate, 0.1% SDS) containing 1 mM phenylmethane sulfonylfluoride and incubated for 30 min on ice, then centrifuged at 4°C with 12000×*g* for 15 min. The supernatant lysates were diluted in 2× or 5×SDS sample buffer, boiled for 5 min. In addition, the cytoplasmic proteins of spleen and total proteins of cultured peritoneal macrophages were also extracted. Protein was quantitated, equal amounts of protein from each sample were separated on SDS-polyacrylamide gel by electrophoresis, and then transferred to a nitrocellulose membrane (Millipore, Billerica, MA, USA). The membrane was blocked with TBST buffer (20 mM Tris–HCl, 137 mM NaCl, and 0.1% Tween 20, pH 7.5) containing 5% (w/v) non-fat dry milk at room temperature for 1 h. Then, the membranes were washed twice with the Tris-buffered saline and incubated overnight at 4°C with the primary antibody anti-IκBα,anti-NF-κB p65, anti-ERK,anti-p38 MAPK, anti-JNK, anti-IRF3, anti-Tyk2, anti-STAT1, anti-p-IκBα,anti- p-ERK,anti-p-p38 MAPK, anti-p-JNK, anti-p-IRF3, anti-p-Tyk2, anti-p-STAT1or anti-GAPDH, followed by incubation with a horseradish peroxidase- conjugated secondary antibody for 1 h at room temperature. Afterwards, the protein bands were exposed to Kodak X-Omat film with an enhanced chemiluminescence reagent (Thermo Scientific, Rockford IL, USA). The band intensities were determined by densitometry.

### Measurement of NF-κB Activity

NF-κB activity was determined using Cayman’s NF-κB (p65) Transcription Factor Assay kit (Cayman chemical company, Ann Arbor, Michigan, USA), a sensitive ELISA method for detecting NF-κB p65 DNA binding activity in nuclear extracts, according to the manufacturer’s protocol. Briefly, nuclear proteins of spleen and peritoneal macrophages (1×10^7^) were isolated using Cayman’s Nuclear Extraction kit (Item No. 10009277, Cayman chemical company, Ann Arbor, Michigan, USA) and quantitated, the equal amount of nuclear protein was mixed with transcription factor binding buffer and then applied to the sample well of a 96-well plate covered with specific double stranded DNA sequence containing NF-κB response element. After incubation overnight at 4°C without agitation, the wells were washed and incubated with NF-κB p65 primary antibody (100 µl) at room temperature for 1 h without agitation, followed by horseradish peroxidase-conjugated secondary antibody (100 µl) at room temperature for 1 h. Each well was washed again and treated with developing solution for 45 min at room temperature. Then, the stop solution (100 µl) was added, absorbance was determined at a wavelength of 450 nm. A positive control for NF-κB p65 activation was also used for this assay and data were normalized to nuclear protein from control mice or peritoneal macrophages.

### Real-time Reverse Transcription-polymerase Chain Reaction (RT-PCR) Assay

The total cellular RNA was isolated using the Trizol protocol (Invitrogen, Carlsbad, CA, USA) from the spleen obtained 1 h after LPS challenge, and cDNA was synthesized using SuperScript (Invitrogen). The primer sequences are listed as follow: IFN-β: forward-5′-AAGCAGCTCCAGCTCCAAGAA-3′, reverse-5′-TTGAAGTCC GCCCTGTAGGTG -3′; IP-10: forward - 5′-TGAATCCGGAATCTAAGACCATC AA-3′, reverse - 5′-AGGACTAGC CATCCACTGGGTAAAG -3′; GAPDH: forward - 5′-TCACCACCATGGAGAAGGC-3′, reverse - 5′-GCTAAGCAGTTGGTGGTGCA -3′. Real time RT-PCR was then performed with the LightCycler® 480 Instrument using SYBR Green I reaction mixture. The amplification program include an initial denaturation at 95°C for 5 min and 45 cycles each consisting of denaturation at 95°C for 10 s, annealing at 60°C for 10 s, and extension at 72°C for 10 s. Relative quantifications of IFN-β and IP-10 gene expression were calculated using the melting curve analysis. The results are expressed as the fold increase over controls in ratio of each target gene to GAPDH.

### Statistical Analysis

Data are presented as mean ± SEM. Significance was determined either with a Student’s t-test or with one-way ANOVA followed by a multiple-comparison test (SPSS 12.0). Mortality was analyzed using Kaplan-Meier survival analysis and compared by log-rank test. The chi-square test was used for determining the significance of differences in survival rate at indicated time point between LPS and drug-treated groups. Values of *p* less than 0.05 were considered to be significant.

## Results

### Ber Significantly Increased the Survival Rate after LPS-induced Sepsis, Which was Enhanced by Y

**Figure 1:**
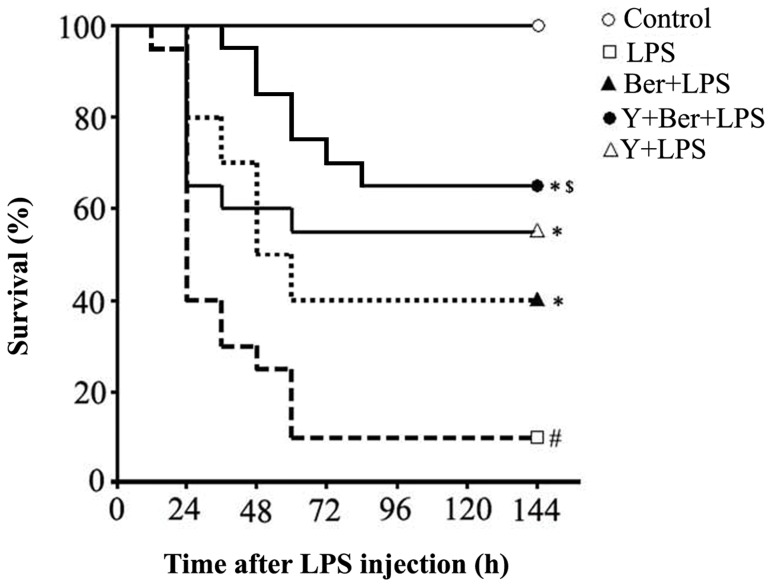
Pretreatment with Ber and yohimbine (Y) protects mice against mortality induced by LPS. Mice were administered intragastrically with Ber (50 mg/kg) or/and Y (2 mg/kg) once a day for 3 days. LPS (20 mg/kg) or normal saline was injected intraperitoneally at 1 h after last intragastrical treatment. The survival rate was evaluated every 12 h for 6 days. Statistical analysis was performed using the log-rank test. *n* = 20 for each group. ^#^ *P<*0.01 compared with control group; *P<0.05 compared with LPS group; *^$^P*<0.05 compared with Ber+LPS group.

In this study, mice were challenged with 20 mg/kg LPS, twelve of twenty mice died in the first 24 h after LPS administration. As shown in Figure 1, the mortality rate reached 90% at 144 h after LPS insult in LPS group. In contrast, in the Ber+LPS, Y+LPS and Y+Ber+LPS groups, the mortality rate was 60%, 45% and 35%,respectively. Oral Ber, Y or Ber in combination with Y pretreatment significantly increased the survival rate after LPS -induced sepsis. Moreover, a combination of Ber and Y – pretreated mice displayed a significantly higher 6-day survival rate (65%) and longer survival time compared with Ber-pretreated animals (40%) after LPS challenge. All normal saline-treated control mice survived in control, Ber, Y+Ber and Y group (data not shown).

### Effects of Ber and Y on Liver and Kidney Injuries in LPS-challenged Mice

**Figure 2:**
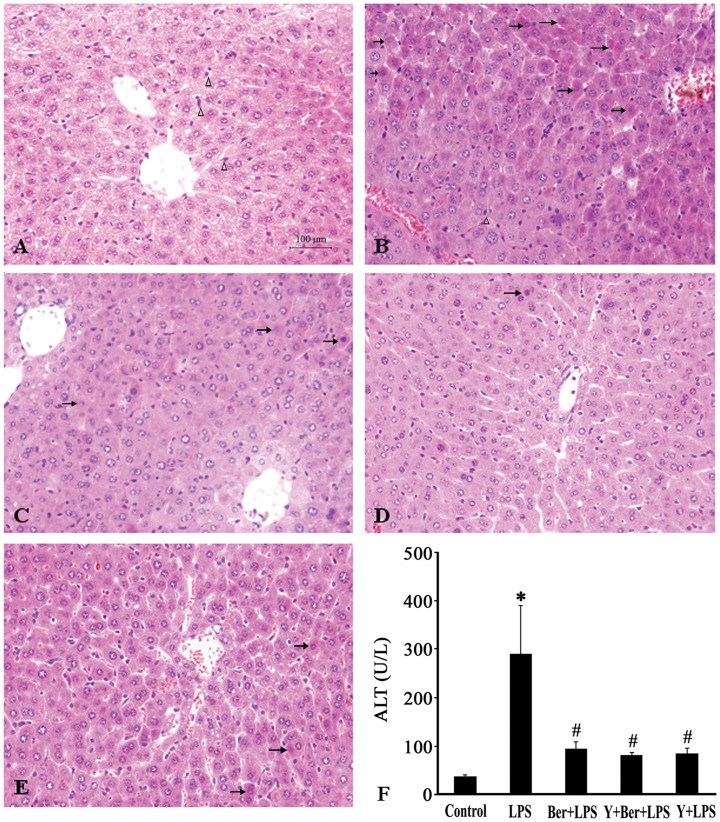
Representative photomicrographs of liver (H&E staining) and plasma ALT levels 12 h after LPS injection. Arrows indicate hepatocyte degeneration and necrosis, triangles indicate Kupffer cells. A: control; B: LPS group; C: Ber+LPS group; D: Y+Ber+LPS group; E: Y+LPS group; F: Plasma ALT level. *n* = 8−10. *P<0.05 compared with control; ^#^ *P<*0.01 compared with LPS group.

**Figure 3:**
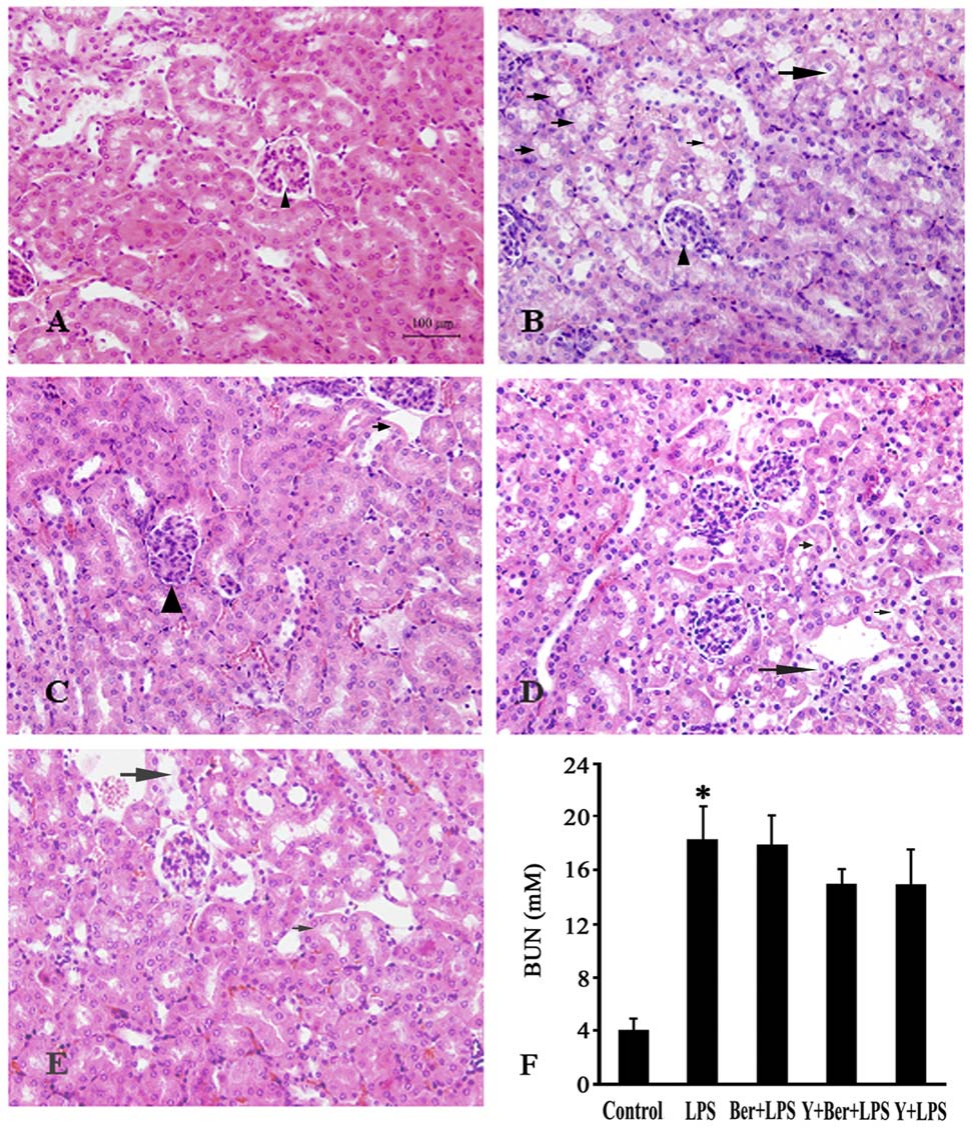
Representative photomicrographs of kidney (H&E staining) and BUN levels 12 h after LPS injection. Arrows indicate degeneration and vacuolization of renal tubular epithelial cells and triangles indicate glomerulus. A: control; B: LPS group; C: Ber+LPS group; D: Y+Ber+LPS group; E: Y+LPS group; F: BUN changes. *n* = 8−10. *P<0.05 compared with control.

To elucidate organ injuries, hepatic and renal histology, plasma indicators for liver function (ALT) and renal function (BUN) were examined 12 h after LPS insult. Figure 2 showed that no pathological changes in the liver samples were found in control. Hepatocyte degeneration and necrosis, hemorrhagic changes, and inflammatory cell infiltration were prominent in the liver at 12 h after LPS injection in LPS group. In contrast, Ber, Y or Ber plus Y pretreatment significantly attenuated these pathological changes induced by LPS. Moreover, LPS caused hepatocellular injury as indicated by significantly elevated plasma ALT levels, and pretreatment with Ber, Y or Ber plus Y also significantly reduced plasma ALT levels in LPS-challenged mice. Renal histopathological examination demonstrated that LPS caused tubular injury, most prominent in the cortex, as manifested by tubular degeneration and vacuolization at 12 h after LPS injection (Figure 3 B), which were not significantly alleviated by Ber or/and Y pretreatment (Figure 3C, 3D and E). The similar findings were observed with regard to plasma levels of BUN, an indicator of renal dysfunction. LPS caused a marked increase in plasma BUN levels in the LPS group at 12 h. The increased levels of BUN were not markedly suppressed by Ber or/and Y pretreatment in LPS-challenged mice (Figure 3F).

### Effects of Ber, Y and Ber Plus Y on Circulating TNF-α, IL-1β, IL-12p40, IFN-γ, IL-10 and NO Concentrations in LPS-challenged Mice

**Figure 4:**
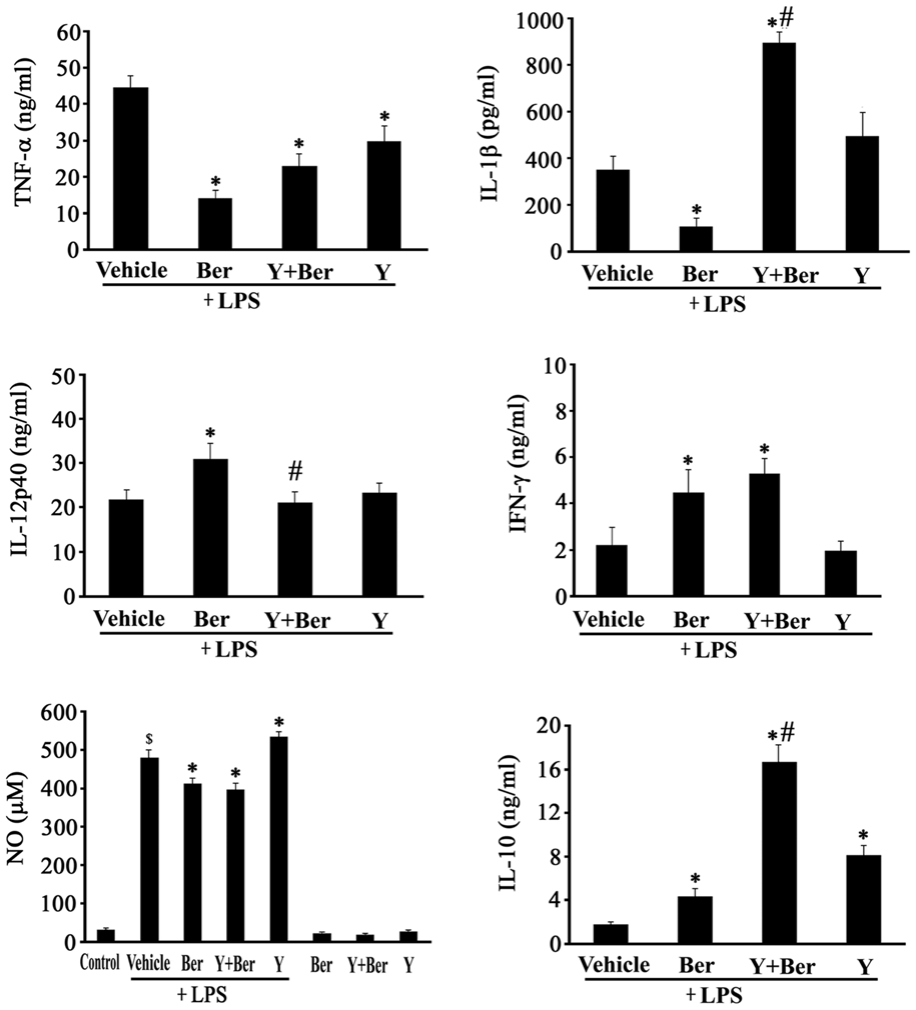
Effects of Ber or/and Y on plasma cytokine and NO levels in LPS-treated mice. TNF-α, IL-1β and IL-10 were measured at 2 h, IL-12p40 and IFN-γ were detected at 4 h, and NO at 8 h after LPS or normal saline injection. n = 8−10. ^$^ *P*<0.05 compared with control, *P<0.01 compared with LPS group,^ #^ *P*<0.01 compared with Ber+LPS group or Y+LPS group.

LPS-induced biological effects, such as shock, multiple organ dysfunction and lethality, are mediated by strong inflammatory responses. To observe the effects of Ber and Y on LPS-induced inflammatory responses, plasma levels of TNF-α, IL-1β, IL-12p40, IFN-γ, IL-10 and NO were analyzed. At 2 h after LPS insult, plasma concentrations of TNF-α, IL-1β and IL-10 elevated significantly in LPS group compared to control. Ber pretreatment reduced plasma TNF-α and IL-1β levels, and enhanced IL-10 production in LPS-challenged mice. Y pretreatment decreased plasma TNF-α concentration and augmented IL-10 production, but failed to attenuate IL-1β levels in LPS-challenged mice. Ber plus Y pretreatment also decreased plasma TNF-α concentration and augmented IL-10 production in LPS-challenged mice. However, Ber plus Y pretreatment significantly promoted LPS-induced IL-1β production. Especially, plasma level of IL-10, an anti-inflammatory cytokine, was markedly higher in Y+Ber+LPS group than that in Ber+LPS group and Y+LPS group. At 4 h after LPS administration, LPS elevated the plasma levels of IL-12p40 and IFN-γ in mice. Ber pretreatment further enhanced IL-12p40 and IFN-γ production, Y pretreatment did not markedly affect plasma IL-12p40 and IFN-γ levels in LPS-challenged mice. However, Ber plus Y pretreatment promoted LPS-induced IFN-γ production, did not markedly altered plasma IL-12p40 level in LPS-challenged mice. Plasma level of IL-12p40 was markedly lower in Y+Ber+LPS group than that in Ber+ LPS group. At 8 h after LPS injection, the plasma level of NO increased in LPS group compared to control. Both Ber and Ber plus Y suppressed LPS-induced NO production. In contrast, Y pretreatment promoted LPS-stimulated NO production (Figure 4).

### Ber, Y and Ber Plus Y All Attenuated LPS-induced NF-κB Activation as well as IκBα, JNK and ERK Phosphorylation, but not p38MAPK Phosphorylation in Spleen of Mice

**Figure 5:**
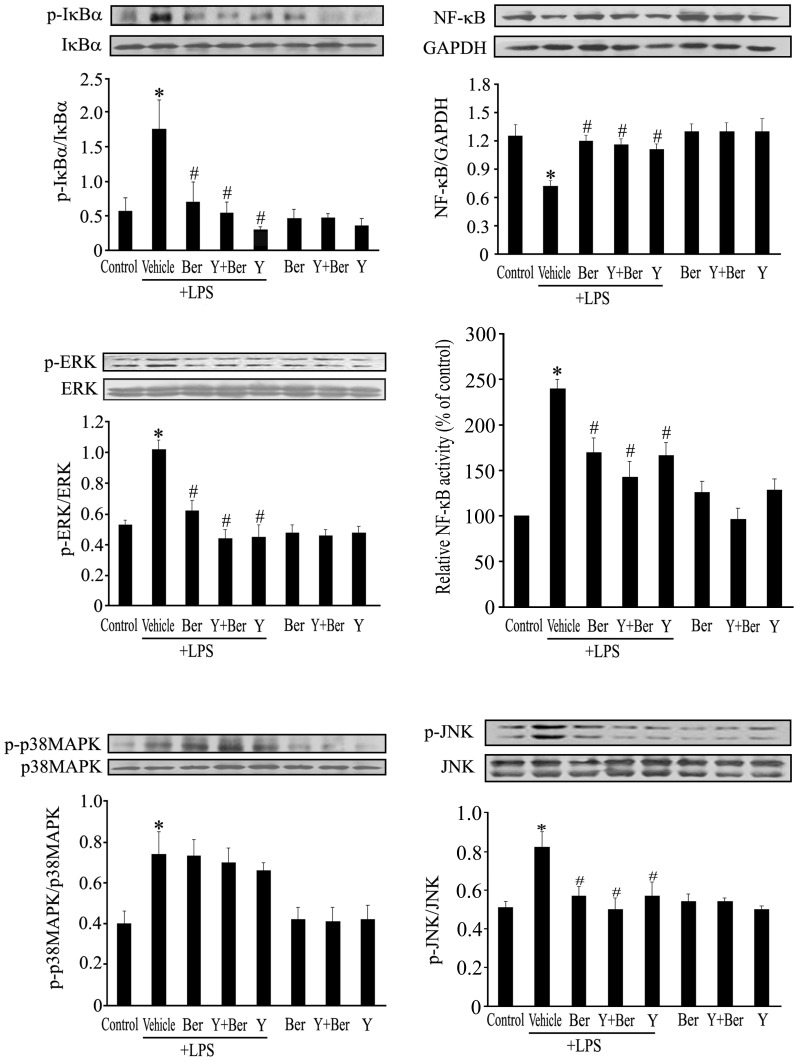
Effects of Ber or/and Y on IκBα, ERK, JNK and p38 MAPK phosphorylation as well as NF-κB activation in spleen of LPS-challenged mice. Spleen was removed 1 h after LPS or normal saline injection, extraction of total protein was analyzed for the presence of total and phosphorylated forms of IκBα, ERK, JNK and p38 MAPK using Western blot, cytoplasmic extracts were subjected to Western blot analysis for determining NF-κB p65 level, nuclear extracts were used to measure the NF-κB activity using NF-κB p65 transcription factor assay kit, and NF-κB activity was normalized to control. *n* = *5−6*. *P<0.05 compared with control group; ^#^ *P*<0.05 compared with LPS group.

It is well known that LPS stimulates inflammatory cytokine expression via MyD88-dependent NF-κB and MAPK activation. We investigated the effects of Ber or/and Y on LPS-stimulated NF-κB activation, IκBα, JNK, ERK and p38MAPK phosphorylation in spleen of mice. As shown in Figure 5, LPS insult increased IκBα, JNK, ERK and p38MAPK phosphorylation in spleen at 1 h after LPS injection. Pretreatment with Ber, Y or Ber plus Y significantly attenuated IκBα, JNK and ERK phosphorylation, but not p38MAPK phosphorylation. In addition, cytoplasmic NF-κB p65 level of spleen at 1 h after LPS challenge in LPS group was lower than that in control, whereas the NF-κB activity of the nuclear fraction in spleen at 1 h after LPS challenge significantly increased in LPS group compared with control. Pretreatment with Ber, Y or Ber plus Y markedly increased cytoplasmic NF-κB p65 level and reduced NF-κB activity of the nuclear protein in spleen at 1 h after LPS challenge in LPS-treated mice (Figure 5).

### Y Enhanced the Inhibitory Effect of Ber on IP-10 Expression through Suppressing MyD88-independent Signal Pathway in LPS-challenged Mice

**Figure 6:**
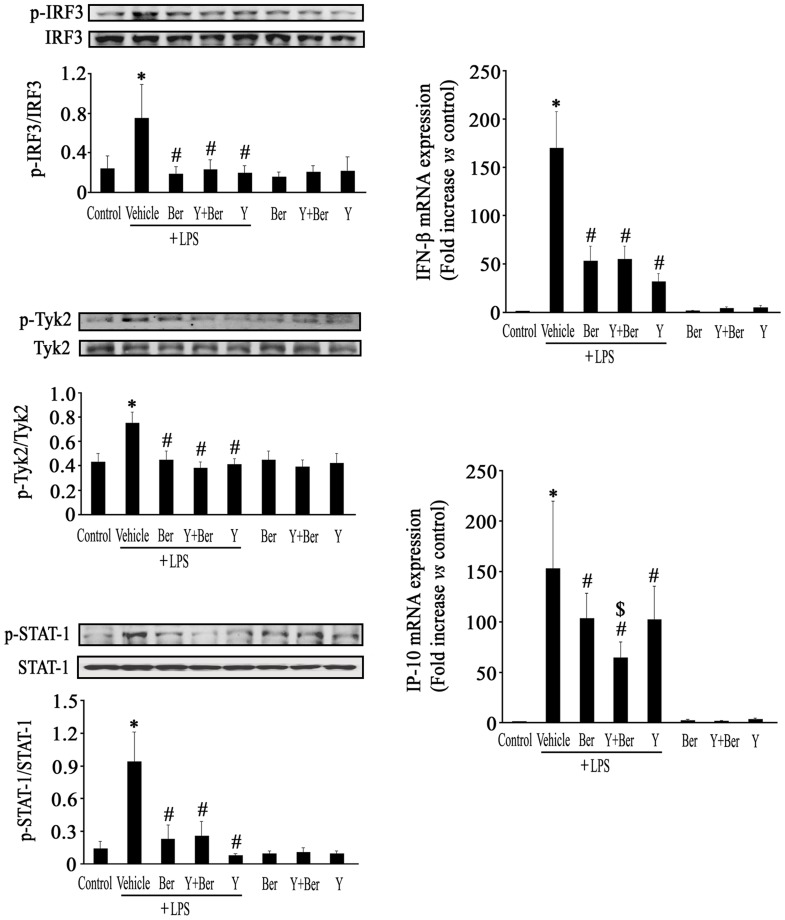
Ber and Y inhibit LPS-induced MyD88-independent signal pathway activation in spleen of mice. Phosphorylation of IRF3, Tyk2 and STAT1 were assessed by Western blot. The expression of IFN-β and IP-10 mRNA were detected by real-time RT-PCR. *n* = *6−8*. *P<0.05 compared with control group; *^#^P*<0.05 compared with LPS group. ^$^ *P*<0.05 compared with Ber+LPS group.

LPS activates not only MyD88-dependent signal pathway, but also MyD88-independent signal pathway. We further examined the effects of Ber, Y and Ber plus Y on LPS-stimulated MyD88-independent signal pathway activation in spleen of mice. The results demonstrated that LPS induced IRF3, TyK2 and STAT1 phosphorylation, as well as IFN-β and IP-10 mRNA expression in spleen of mice at 1 h after LPS challenge, all of which were blocked by Ber, Ber plus Y or Y pretreatment. In particular, IP-10 mRNA expression was significantly lower in Y+Ber+LPS group than that in Ber+LPS group (Figure 6).

### Y Enhanced the Inhibitory Effect of Ber on TNF-α Production and Ber Plus Y Promoted IL-10 Production in LPS-activated Macrophages

**Figure 7:**
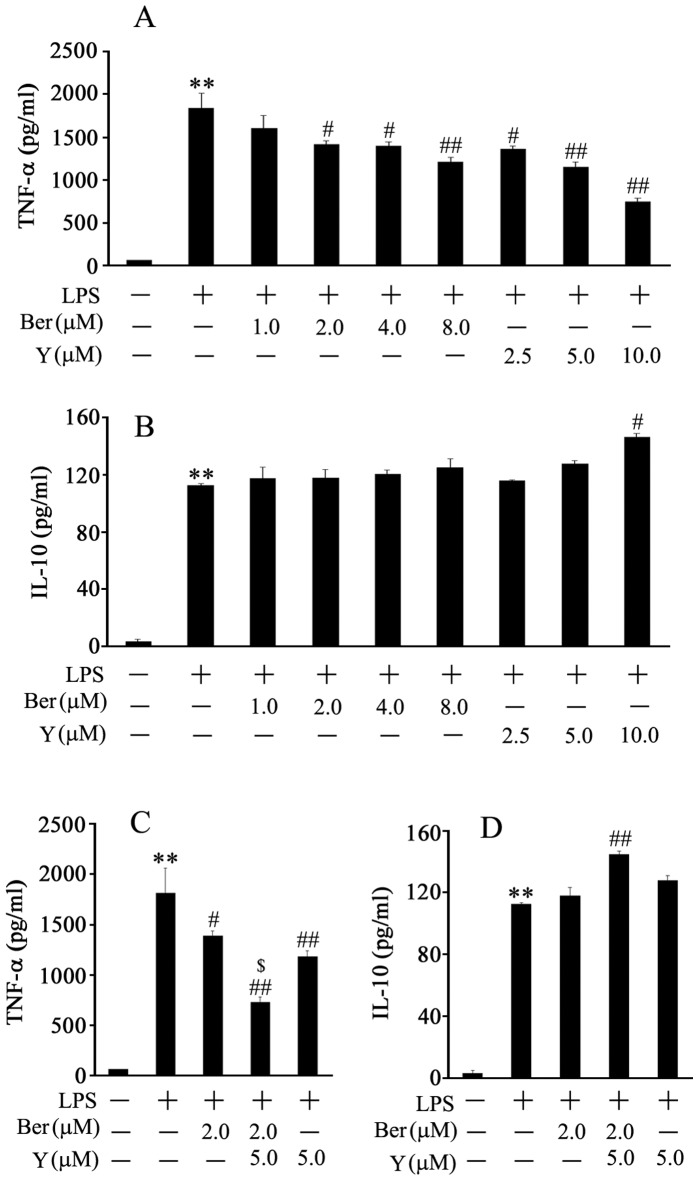
Effects of Ber and Y on LPS-induced TNF-α and IL-10 production in cultured peritoneal macrophages. Mouse peritoneal macrophages were isolated and incubated with vehicle, Ber or/and Y for 2 h and then with vehicle or LPS (100 ng/ml) for another 6 h. TNF-α and IL-10 concentrations in culture supernatants were examined by ELISA, data shown represent the mean of four independent experiments. (A) Ber (1.0–8.0 µM) and Y (2.5–10.0 µM) inhibit LPS-stimulated TNF-α production in a dose-dependent manner. (B) Y at a dose of 10.0 µM enhances LPS-induced IL-10 production. (C) Y promotes the inhibitory effect of Ber on LPS-stimulated TNF-α production. (D) A combination of Ber and Y elevates IL-10 production in LPS-treated macrophages. **P<0.01 compared with control group; ^#^ *P*<0.05, ^##^ *P*<0.01 compared with LPS group. ^$^ *P*<0.05 compared with Ber+LPS or Y+LPS group.

The activation of macrophages by LPS is an important event during endotoxemia, LPS activates macrophages to produce cytokines such as TNF-α and IL-10 [7]. Therefore, mouse peritoneal macrophages were isolated to further study the effects of Ber and Y on TNF-α and IL-10 production. We found that pretreatment with Ber or Y significantly suppressed TNF-α production in LPS-activated macrophages in a dose-dependent manner and Y markedly enhanced the inhibitory effect of Ber on LPS-induced TNF-α production (Figure 7A and C). Ber at doses of 1.0, 2.0, 4.0 and 8.0 µM did not significantly promote IL-10 release at 6 h at LPS exposure in LPS-activated macrophages. Y at a dose of 10 µM enhanced LPS-induced IL-10 production. Although Ber (2.0 µM) or Y (5.0 µM) alone did not elevate IL-10 production in LPS-activated macrophages, Ber (2.0 µM) plus Y (5.0 µM) significantly increased LPS-induced IL-10 production (Figure 7B and D). In addition, Ber, Y or/and LPS at indicated concentrations did not significantly affect viability of macrophages (data not shown).

### Ber, Y and Ber Plus Y All Suppressed LPS-induced NF-κB Activation, IκBα, JNK, ERK and IRF3 Phosphorylation, but not p38MAPK Phosphorylation in Macrphages

**Figure 8:**
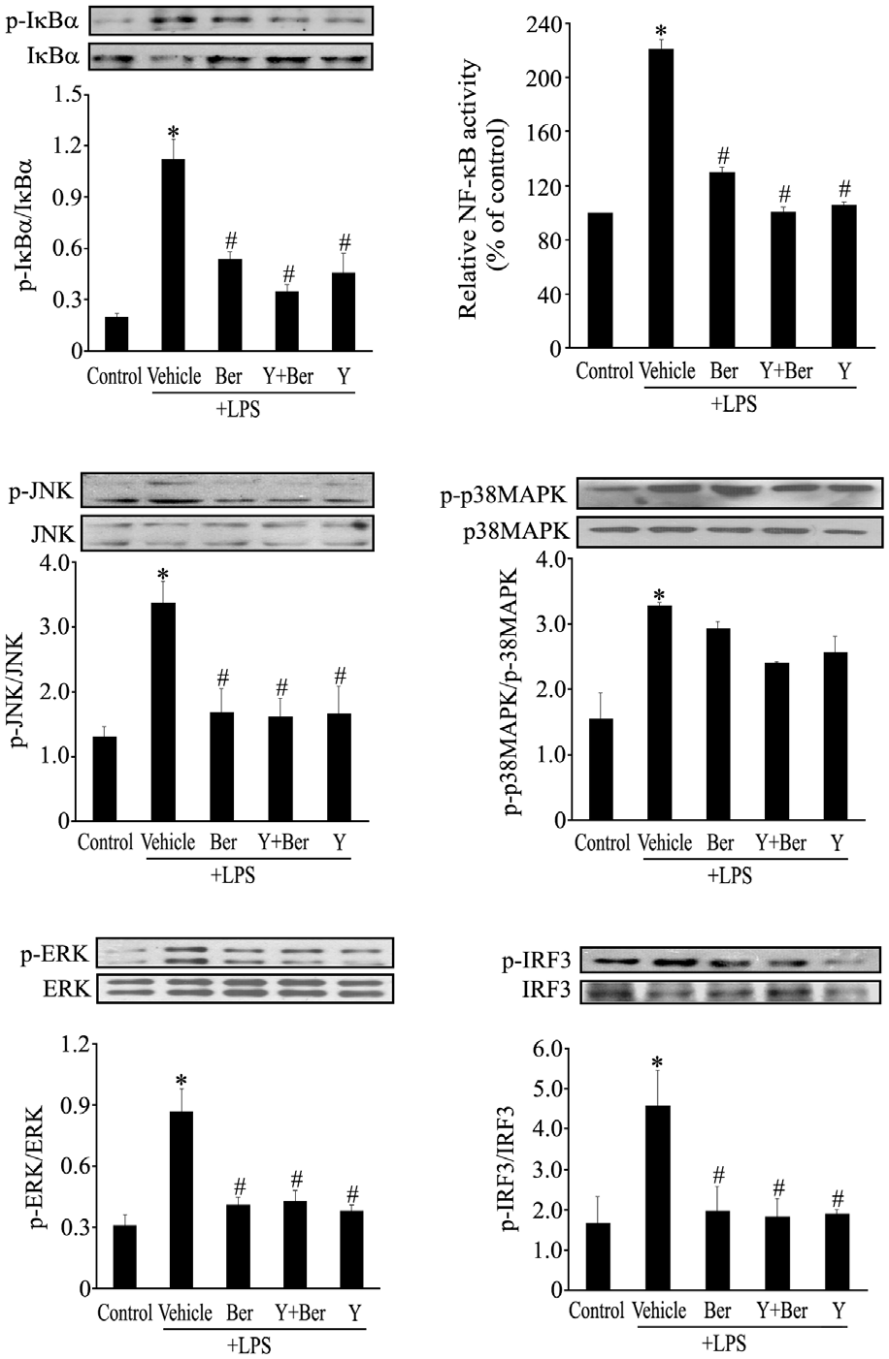
Ber and Y suppress IκBα, ERK, JNK and IRF3 phosphorylation as well as NF-κB activation, but not p38 MAPK phosphorylation in LPS-treated macrophages. Mouse peritoneal macrophages were incubated with vehicle, Ber (2.0 µM), Ber (2.0 µM)+Y (5.0 µM) or Y (5.0 µM) for 2 h and then treated with LPS (100 ng/ml) for another 1 h. Whole cell lysates were examined for the presence of total and phosphorylated form of IκBα, ERK, JNK, p38 MAPK and IRF3 using Western blotting. Data in graph are presented as the mean of the ratio of phosphorylated protein to total protein. Nuclear extracts were also used to measure the NF-κB activity using NF-κB p65 transcription factor assay kit, and NF-κB activity was normalized to control macrophages. *n* = 3−4. *P<0.05 compared with control group; *^#^P*<0.05 compared with LPS group.

To further study the effects of Ber and Y on MyD88-dependent and independent signal pathways in LPS-activated macrophages, we examined the NF-κB activation as well as IκBα, JNK, ERK, p38 MAPK and IRF3 phosphorylation in macrophages at 1 h after LPS exposure. The results demonstrated that LPS stimulation for 1 h significantly increased IκBα, JNK, ERK, p38 MAPK and IRF3 phosphorylation, pretreatment with Ber, Y or Ber plus Y significantly suppressed LPS-induced IκBα, JNK, ERK and IRF3 phosphorylation, but not p38 MAPK phosphorylation in macrophages. In addition, LPS significantly upregulated the NF-κB activity of nuclear fraction in macrophages at 1 h after LPS challenge, which was inhibited by Ber or/and Y pretreatment (Figure 8).

## Discussion

Our previous study demonstrated that oral Ber (50 mg/kg) decreased mortality in mice challenged with LPS, which was enhanced by Y (2 mg/kg) administered intraperitoneally 30 min before Ber treatment [12]. However, the mechanism of these actions is unclear. This study confirmed that oral Ber (50 mg/kg) in combination with Y (2 mg/kg) improved survival in LPS-treated mice, and oral Y also enhanced the protection of Ber against LPS-induced lethality. We also observed that there was no significant difference in survival rate of LPS-challenged mice between Y and Ber plus Y pretreatment groups. This causes the rationale behind the combined treatment with Ber plus Y not to be better established. However, our previous investigation demonstrated that a combination of Ber and Y administered 2 h after CLP improved survival in septic mice with better efficacy than Ber or Y alone and Ber or Y alone did not reduced the CLP-induced mortality in mice. Therefore, the mechanisms by which Y enhances the protection of Ber against endotoxemia deserve to be further investigated. Because the lethality induced by LPS is associated with organ failure, we examined the pathology and function of liver and kidney in LPS-challenged mice. The results revealed that Ber, Y or Ber plus Y pretreatment attenuated liver injury, but not renal injury in LPS-challenged mice. Miksa, et al found that activation of α_2_-adrenergic receptors by norepinephrine in Kupffer cells played an important role in LPS-induced liver damage [14]. Thus, blockage of α_2_-adrenergic receptors by Y can inhibit hepatic injury during endotoxemia. Our recent study demonstrated that Y (2 mg/kg) prevented LPS-induced cardiac dysfunction [15]. Although the mechanisms responsible for protection of Y against LPS-induced cardiac injury remain to be further investigated, these findings indicates that Y enhances the protective effect of Ber against LPS-induced lethality in mice, at least in part, via preventing liver and cardiac injury during endotoxemia. In addition, it was reported that Y pretreatment failed to reduce LPS-induced lung and intestinal injuries [12], [16], this study observed that Y inhibited LPS-induced liver, but not renal damage. These observations also suggest that the role of α_2_-adrenergic receptor activation by norepinephrine is different in different organ dysfunction during endotoxemia.

It is well known that a progressive release of cytokines and other inflammatory mediators, including TNF-α, IL-1β, IL-12p40, IFN-γ and NO, involve the trigger of multiple organ dysfunction during endotoxemia [17]. Some studies demonstrated that plasma TNF-α, IL-1β and IL-10 transiently increased and peaked at 2 h, the levels of plasma IL-12p40 and IFN-γ peaked 4 h, but plasma NO levels peaked 8 h after LPS challenge. Thus, we detected plasma TNF-α, IL-1β, IL-12p40, IFN-γ and NO concentrations at the indicated time point after LPS treatment [18], [19], [20]. The results showed that Ber reduced plasma TNF-α, IL-1β and NO levels, enhanced IL-12p40 and IFN-γ production in LPS-challenged mice. Y decreased plasma TNF-α concentration and augmented NO production, while Ber plus Y decreased plasma TNF-α and NO concentrations and promoted IL-1β and IFN-γ production in LPS-challenged mice. Although Ber, Y and Ber plus Y all decreased mortality rate in endotoxemic mice, their effects on the profile of inflammatory mediator production induced by LPS are different. The previous studies demonstrated that none of mice, deficient in TNF receptor, IL-1 or inducible NO synthase, survived a high-dose LPS insult [21], [22], [23], suggesting that none of single cytokine or inflammatory mediator is sufficient to produce the complete spectrum of toxic effects induced by LPS. Thus, these findings indicates that changes of the above single cytokine did not fully explain the protection of Ber or/and Y against LPS-induced lethality.

This study further observed that Ber, Y and Ber in combination with Y all inhibited LPS-stimulated NF-κB activation as well as IκBα, JNK and ERK phosphorylation, this may be responsible for the mechanisms underlying the inhibition of LPS-induced TNF-α production by these drugs. However, the present study also observed that LPS-induced NF-κB activation, which is involved in IL-1β expression, was suppressed by treatment with Ber or/and Y, Ber decreased plasma IL-1β level, but Ber plus Y significantly increased plasma IL-1β level in LPS-challenged mice. It is well known that IL-1β production and maturation are tightly controlled by caspase-1 [24]. Thus, the mechanisms underlying increased IL-1β production by Ber plus Y in LPS-challenged mice might be associated with caspase-1. This remains to be further examined.

Kang et al. found that Ber stimulated IL-12 p40 production via activating p38 MAPK in mouse macrophages, which was blocked by Y pretreatment, and Ber significantly enhanced IL-12 p40 production when combined with LPS [11]. Consistent with this observation, we also found Ber promoted IL-12 p40 production in LPS-challenged mice, which was inhibited by Y. It has been demonstrated that IL-12 upregulates IFN-γ regulation [25]. Thus, in this study, increased IFN-γ production by Ber in LPS-challenged mice may be related with enhanced IL-12 production, but the mechanism by which Ber plus Y promoted IFN-γ production in endotoxemic mice needs to be further investigated. On the other hand, exogenously administered IL-10, an anti-inflammatory cytokine, was found to successfully counteract the lethal effects of LPS [26], conversely, anti-IL-10 monoclonal antibody treatment rendered mice more sensitive to LPS [27]. In the present study, we observed that Ber, Y and Ber plus Y all enhanced LPS-stimulated IL-10 production in mice. In addition, plasma level of IL-10 was markedly higher in Y+Ber+LPS group than that in Ber+LPS group and Y+LPS group. Therefore, these results suggest that Y may enhance the protection of Ber against LPS-induced lethality via promoting IL-10 production. Although we found that Ber, Ber plus Y and Y did not affected LPS-stimulated p38 MAPK activation, which has been demonstrated to be involved in the synergistic IL-10 induction by LPS and other inducers [28], the mechanism by which Y promoted IL-10 production caused by Ber in LPS-challenged mice remains to be further examined.

Except for MyD88-dependent signal pathway, LPS also activates IRF3 and stimulates expression of IFN-β, in turn causing phosphorylation of Tyk2 and STAT1 and subsequently inducing IP-10 expression in a MyD88-independent manner [7], [8], [29]. A series of investigations have demonstrated that mice lacking IRF3, IFN-β, Tyk2, or Stat1 were resistant to LPS-induced lethality [8], [30], [31]. In this study, we further observed that Ber, Ber plus Y or Y pretreatment suppressed LPS-induced IRF3, TyK2 and STAT1 phosphorylation, as well as IFN-β and IP-10 mRNA expression in mice at 1 h after LPS challenge. Especially, Y enhanced the inhibitory effect of Ber on IP-10 mRNA expression in LPS-challenged mice. These findings indicated that Y enhanced protection of Ber against endotoxemia partly via suppressing MyD88-independent signaling activation due to inhibition of IRF3 phosphorylation induced by LPS. However, the mechanism responsible for the suppression of LPS-induced IRF3 phosphorylation by Ber and Y is unclear. Recently, noradrenaline was found to inhibit expression of IP-10 in the central nervous system following a systemic LPS challenge [32], we also observed that Y could increase the plasma concentration of noradrenaline during endotoxemia (data not shown). It is possible that Y reduces MyD88-independent pathway activation in endotoxemic mice via noradrenaline.

Moreover, activation of macrophages by LPS has been demonstrated to be an important event during endotoxemia [7]. To further understand the mechanisms underlying the enhanced protection of Ber against endotoxemia by Y, we investigated the effects of Ber and Y on MyD88-dependent and independent pathways as well as cytokine production in LPS-treated mouse peritoneal macrophages. The results confirmed that pretreatment with Ber, Y or Ber plus Y significantly suppressed LPS-induced NF-κB activation as well as IκBα, JNK, ERK and IRF3 phosphorylation, but not p38 MAPK phosphorylation. In addition, Y markedly enhanced the inhibitory effect of Ber on LPS-induced TNF-α production and Ber plus Y promoted LPS-induced IL-10 production in macrophages. These findings indicate that enhanced protection of Ber against LPS-induced lethality by Y may be, at least in part, due to inhibition of IκBα, JNK, ERK and IRF3 phosphorylation and upregulation of IL-10 in macrophages.

In summary, the present study provides evidence that orally-administered Y enhances the protective effect of Ber against LPS-induced lethality in mice via attenuating liver injury, upregulating IL-10 production and suppressing phosphorylation of IκBα, JNK, ERK and IRF3 in macrophages. These findings indicate that Ber in combination with Y can function as a potent immunomodulator that regulates MyD88-dependent and independent signal pathways, and thus preventing the underlying pathological process that eventually results in death during endotoxemia. Because Ber in combination with Y administered 2 h after CLP has been found to protect mice against sepsis-induced lethality in a CLP - induced model of sepsis, a combination of Ber and Y may be of interest for clinical use.
